# Time and event-specific deep learning for personalized risk assessment after cardiac perfusion imaging

**DOI:** 10.1038/s41746-023-00806-x

**Published:** 2023-05-01

**Authors:** Konrad Pieszko, Aakash D. Shanbhag, Ananya Singh, M. Timothy Hauser, Robert J. H. Miller, Joanna X. Liang, Manish Motwani, Jacek Kwieciński, Tali Sharir, Andrew J. Einstein, Mathews B. Fish, Terrence D. Ruddy, Philipp A. Kaufmann, Albert J. Sinusas, Edward J. Miller, Timothy M. Bateman, Sharmila Dorbala, Marcelo Di Carli, Daniel S. Berman, Damini Dey, Piotr J. Slomka

**Affiliations:** 1 Departments of Medicine (Division of Artificial Intelligence in Medicine), Imaging, and Biomedical Sciences, Cedars-Sinai Medical Center, Los Angeles, CA USA; 2 Department of Interventional Cardiology and Cardiac Surgery, Collegium Medicum, University of Zielona Góra, Zielona Góra, Poland; 3 Department of Nuclear Cardiology, Oklahoma Heart Hospital, Oklahoma City, OK USA; 4 Department of Cardiac Sciences, University of Calgary and Libin Cardiovascular Institute, Calgary, AB Canada; 5 Institute of Cardiovascular Science, University of Manchester, Manchester, UK; 6 Department of Cardiology, Manchester Heart Institute, Manchester Royal Infirmary, Manchester University NHS Foundation Trust, Manchester, UK; 7 Department of Interventional Cardiology and Angiology, Institute of Cardiology, Warsaw, Poland; 8 Department of Nuclear Cardiology, Assuta Medical Centers, Tel Aviv, Israel; 9 Division of Cardiology, Department of Medicine and Department of Radiology, Columbia University Irving Medical Center and NewYork-Presbyterian Hospital, New York, NY USA; 10 Oregon Heart and Vascular Institute, Sacred Heart Medical Center, Springfield, OR USA; 11 Division of Cardiology, University of Ottawa Heart Institute, Ottawa, ON Canada; 12 Department of Nuclear Medicine, Cardiac Imaging, University Hospital Zurich, Zurich, Switzerland; 13 Section of Cardiovascular Medicine, Department of Internal Medicine, Yale University School of Medicine, New Haven, CT USA; 14 Cardiovascular Imaging Technologies LLC, Kansas City, MO USA; 15 Department of Radiology, Division of Nuclear Medicine and Molecular Imaging, Brigham and Women’s Hospital, Boston, MA USA

**Keywords:** Machine learning, Predictive medicine, Computational models, Image processing

## Abstract

Standard clinical interpretation of myocardial perfusion imaging (MPI) has proven prognostic value for predicting major adverse cardiovascular events (MACE). However, personalizing predictions to a specific event type and time interval is more challenging. We demonstrate an explainable deep learning model that predicts the time-specific risk separately for all-cause death, acute coronary syndrome (ACS), and revascularization directly from MPI and 15 clinical features. We train and test the model internally using 10-fold hold-out cross-validation (*n* = 20,418) and externally validate it in three separate sites (*n* = 13,988) with MACE follow-ups for a median of 3.1 years (interquartile range [IQR]: 1.6, 3.6). We evaluate the model using the cumulative dynamic area under receiver operating curve (cAUC). The best model performance in the external cohort is observed for short-term prediction – in the first six months after the scan, mean cAUC for ACS and all-cause death reaches 0.76 (95% confidence interval [CI]: 0.75, 0.77) and 0.78 (95% CI: 0.78, 0.79), respectively. The model outperforms conventional perfusion abnormality measures at all time points for the prediction of death in both internal and external validations, with improvement increasing gradually over time. Individualized patient explanations are visualized using waterfall plots, which highlight the contribution degree and direction for each feature. This approach allows the derivation of individual event probability as a function of time as well as patient- and event-specific risk explanations that may help draw attention to modifiable risk factors. Such a method could help present post-scan risk assessments to the patient and foster shared decision-making.

## Introduction

Through recent advances, artificial intelligence (AI) has established an important new paradigm in medical image analysis, potentially enhancing prognostic applications from all cardiovascular imaging modalities. However, there are challenges in how risks are conveyed to both physicians and patients to facilitate the best and most appropriate preventative strategies. A single metric of all risks combined is perhaps less useful than a diverse map of individual risks, their timeline, and influencing factors^1^.

Myocardial perfusion imaging (MPI) is a well-established technique for diagnosing coronary artery disease. Although the primary purpose of MPI is the assessment of the flow-limiting coronary artery disease, it is often used for risk stratification^2^. Prognostic risk assessment has been based on the distribution and burden of ischemia detected, usually combined with a composite score of clinical risk factors or inferred from an expert clinical impression—while this has proven to be a generally successful model from a statistical viewpoint, it is arguably relatively crude at the individual patient level. In the status quo, although a patient may be informed that they are at high risk for an adverse event, they are left with less information about what type of event, or within what timeframe can be anticipated, and this can be more unnerving than productive. While researchers have successfully used AI to facilitate, quantify, and automate several aspects of the conventional imaging workflow for diagnosing of disease^3^, the efforts applied to prognostic interpretation lack time-specific or event-specific prediction^4^. To date, proposed predictive AI models for cardiovascular image interpretation do not differentiate between the possible adverse events and oversimplify the predicted risk to a single numeric value despite the richness and depth of the source data^5^. Compounding this issue is the significant heterogeneity of the definitions of composite end-points, such as major adverse cardiovascular events (MACE) in prior clinical studies^6^.

**Fig. 1:**
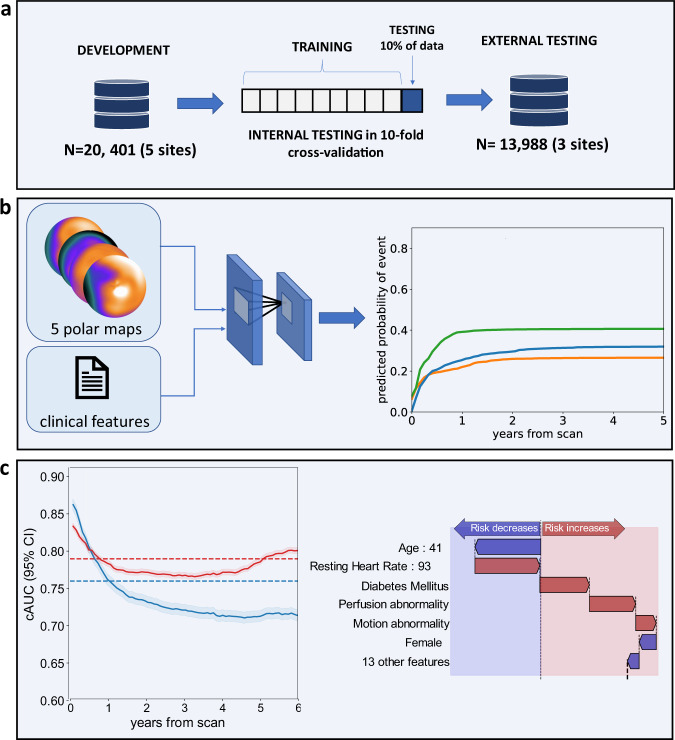
Deep learning enabled time-to-event outcome prediction after cardiac imaging - study overview. **a** A time-to-event deep learning model was trained (left) using data from the 5 sites of the REFINE SPECT registry (*n* = 20,401), then tested internally in a 10-fold cross-validation regimen (middle) and tested in 3 external sites (*n* = 13,988) (right); **b** The time-to-event model uses 5 SPECT polar maps and 15 clinical features as inputs (left) and predicts time-dependent probability of death (orange line), ACS (green line), and revascularization (blue line); **c** The performance of the model (left) is analyzed using cumulative dynamic area under the receiver-operating curves (cAUC). Red line represents the time-to-event model and blue line represents perfusion abnormality. The explanation of the prediction is visualized as a waterfall plot with blue arrows representing features that decrease the risk and red arrows representing the features that increase the risk (right); ACS acute coronary syndrome, AUC area under the receiver operating characteristics curve, TPD total perfusion deficit, PCI percutaneous coronary intervention, CI confidence intervals.

To date, no methods are established to predict time-dependent risks of specific event types (such as death or myocardial infarction) from a single model after cardiovascular imaging. In this study, we aimed to create a deep learning model capable of predicting patient and event-specific risk over time directly from combined cardiac perfusion image and clinical data. We also describe methods for visual explanation of these predicted risks over time which can be presented to physicians and patients. This could be applied to patient care by presenting patient-individualized survival curves for specific events and explaining the contribution of risk factors to event risk, potentially leading to patient engagement and tailoring therapy for the prevention of adverse events. The overview of the study is presented in Fig. 1.

## Results

### Population characteristics

The training and internal testing cohort included 20,401 patients followed up for MACE for a median of 4.4 years (interquartile range [IQR]: 3.4, 5.7). All-cause death was observed in 1,396 patients (6.8%) and the median time to death was 2.3 years (IQR: 1.1, 3.7). ACS was observed in 657 patients (3.2%) and the median time to that event was 1.6 years (IQR: 0.6, 3.0). Revascularization was observed in 1,485 cases (7.3%) and the median time to revascularization was 0.6 years (0.1, 2.3). Summary of the clinical characteristics of the derivation cohort is shown in Table 1.

**Table 1:** Characteristics of the REFINE SPECT set (training and internal testing).

	Overall, *N* = 20,401	No events, *N* = 16,863	Acute Coronary Syndrome, *N* = 657	Death, *N* = 1396	Revasc., *N* = 1485	*p* value
Age	64 (56, 73)	63 (55, 72)	67 (59, 76)	72 (64, 81)	66 (59, 74)	<0.001^a^
Male	11,630 (57%)	9,152 (54%)	451 (69%)	853 (61%)	1,174 (79%)	<0.001^b^
BMI	27.3 (24.6, 30.9)	27.3 (24.6, 30.9)	27.3 (24.5, 30.6)	26.6 (23.6, 31.0)	27.6 (25.2, 30.9)	<0.001^a^
Post PCI	3,963 (19%)	2,769 (16%)	271 (41%)	284 (20%)	639 (43%)	<0.001^b^
Post TAVR	46 (0.2%)	25 (0.1%)	5 (0.8%)	15 (1.1%)	1 (<0.1%)	<0.001^b^
Post CABG	1,693 (8.3%)	1,156 (6.9%)	114 (17%)	199 (14%)	224 (15%)	<0.001^b^
Hypertension	12,903 (63%)	10,271 (61%)	517 (79%)	1,039 (74%)	1,076 (72%)	<0.001^b^
Diabetes Mellitus	5,204 (26%)	3,877 (23%)	249 (38%)	529 (38%)	549 (37%)	<0.001^b^
Dyslipidemia	12,890 (63%)	10,341 (61%)	493 (75%)	899 (64%)	1,157 (78%)	<0.001^b^
Family History	5,635 (28%)	4,816 (29%)	181 (28%)	273 (20%)	365 (25%)	<0.001^b^
Stress test type: Exercise	9,721 (48%)	8,576 (51%)	225 (34%)	300 (21%)	620 (42%)	<0.001^b^
Stress test type: Pharmacologic	10,676 (52%)	8,283 (49%)	432 (66%)	1,096 (79%)	865 (58%)	<0.001^b^
Resting Heart Rate	69 (60, 78)	68 (60, 78)	67 (60, 75)	71 (62, 81)	70 (61, 79)	<0.001^a^
Stress heart rate-peak	122 (91, 146)	127 (94, 148)	100 (82, 133)	93 (77, 121)	114 (90, 139)	<0.001^a^
Stress Systolic BP-peak	150 (130, 170)	150 (130, 170)	145 (126, 170)	135 (120, 156)	150 (130, 170)	<0.001^a^
Extent of perfusion abnormality (%)	2 (1, 5)	2 (1, 5)	5 (2, 12)	4 (1, 9)	8 (3, 16)	<0.001^a^

Statistics presented: median (inter quartile range), *n* (%); Statistical tests used:

^a^Kruskal-Wallis rank sum test.

^b^Fisher’s exact test; Abbreviations: *Revasc*. revascularization, *BMI* body mass index, *BP* blood pressure, *CABG* coronary artery bypass grafting, *CABG* coronary artery bypass grafting, *TAVR* transcutaneous aortic valve replacement.

The external testing set included 13,988 patients followed up for MACE for a median of 3.1 years (IQR: 1.6, 3.6). All-cause death was observed in 683 patients (5%) and occurred after a median of 1.5 (IQR: 0.6, 2.5) years from the scan. Acute coronary syndrome (ACS) was observed in 361 (2.5%) of patients after a median of 1.3 years from scan (IQR: 0.5, 2.3) and 918 patients (6.6%) underwent revascularization after a median of 0.1 years from baseline imaging (IQR: 0.03, 1.3). Summary of the clinical characteristics of the external cohort is shown in Table 2.

**Table 2:** Characteristics of the external testing set.

	Overall, *N* = 13,988	No events, *N* = 12,026	Acute Coronary Syndrome, *N* = 361	Death, *N* = 683	Revasc., *N* = 918	*p* value
Age	67 (59, 75)	66 (58, 74)	69 (61, 77)	73 (65, 80)	69 (61, 75)	<0.001^a^
Male	7621 (54%)	6309 (52%)	237 (66%)	414 (61%)	661 (72%)	<0.001^b^
BMI	29 (26, 34)	29 (26, 34)	29 (25, 33)	28 (24, 32)	29 (26, 33)	<0.001^a^
Post PCI	1387 (9.9%)	1030 (8.6%)	101 (28%)	84 (12%)	172 (19%)	<0.001^b^
Post TAVR	9 (<0.1%)	5 (<0.1%)	0 (0%)	0 (0%)	4 (0.4%)	0.009^b^
Past CABG	843 (6.0%)	602 (5.0%)	59 (16%)	64 (9.4%)	118 (13%)	<0.001^b^
Hypertension	8921 (64%)	7477 (62%)	264 (73%)	482 (71%)	698 (76%)	<0.001^b^
Diabetes Mellitus	4023 (29%)	3240 (27%)	151 (42%)	235 (34%)	397 (43%)	<0.001^b^
Dyslipidemia	4635 (33%)	3982 (33%)	161 (45%)	270 (40%)	222 (24%)	<0.001^b^
Family History	2496 (18%)	2228 (19%)	88 (24%)	120 (18%)	60 (6.5%)	<0.001^b^
Stress test type: Exercise	7069 (51%)	6395 (53%)	112 (31%)	146 (21%)	416 (45%)	<0.001^b^
Stress test type: Pharmacologic	6919 (49%)	5631 (47%)	249 (69%)	537 (79%)	502 (55%)	<0.001^b^
Resting Heart Rate	73 (64, 83)	73 (64, 83)	71 (63, 80)	75 (66, 85)	72 (64, 82)	<0.001^a^
Stress heart rate-peak	126 (97, 146)	129 (100, 148)	100 (83, 131)	98 (83, 120)	121 (96, 137)	<0.001^a^
Stress Systolic BP-peak	157 (134, 178)	159 (136, 180)	146 (125, 165)	132 (113, 152)	158 (134, 178)	<0.001^a^
Extent of perfusion abnormality (%)	2.9 (1.1, 6.2)	2.6 (1.0, 5.5)	5.8 (2.2, 12.2)	4.8 (1.8, 10.8)	6.8 (3.3, 13.7)	<0.001^a^

Statistics presented: median (interquartile range), *n* (%); Statistical tests used:

^a^Kruskal-Wallis rank sum test.

^b^Fisher’s exact test; Abbreviations: *Revasc*. revascularization, *BMI* body mass index, *BP* blood pressure, *CABG* coronary artery bypass grafting, *CABG* coronary artery bypass grafting, *TAVR* transcutaneous aortic valve replacement.

### Internal testing

**Fig. 2:**
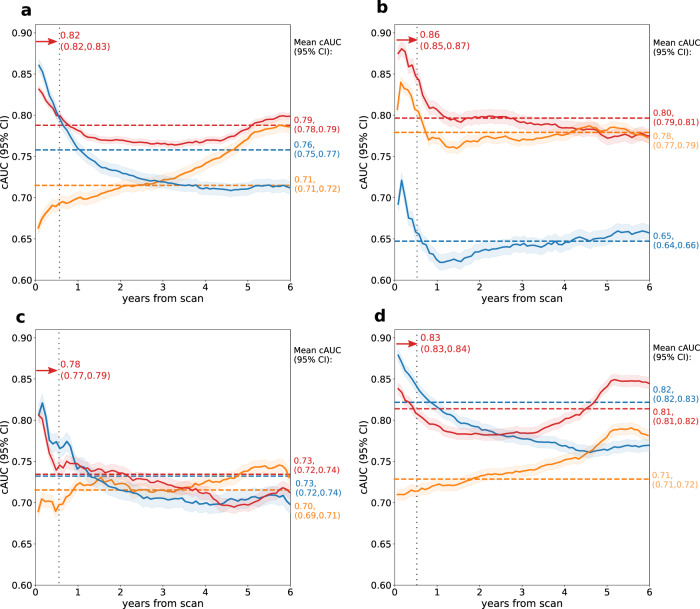
Cumulative dynamic areas under the receiver operating characteristic curve (cAUC) in the internal testing set. The cAUCs are presented for the prediction of any adverse event (**a**) as well as specific events: all-cause death (**b**), acute coronary syndrome (**c**), and revascularization (**d**) in the internal testing dataset. Results were generated separately for each of the 10 testing folds of the cross-validation (*N* = 2041) and then averaged. The red, blue, and orange lines represent cAUC for the time-to-event model, perfusion abnormality, and clinical-only model, respectively. The red, blue, and orange dashed lines represent mean cAUC for the time-to-event model, perfusion abnormality, and clinical-only model, respectively. Shaded areas represent 95% confidence intervals (CI). The red arrows show the cAUC values of time-to-event deep learning averaged over the first six months after the scan (vertical dashed line).

We present the cumulative dynamic area under receiver operating curve (cAUC) for the prediction of any event as well as each of the separate events in Fig. 2. The best performance for the prediction of ACS and all-cause death was observed for short-term prediction – in the first six months after scan, mean cAUC for ACS and all-cause death reached 0.78 (95% confidence interval [CI]: 0.77, 0.79) and 0.86 (95% CI: 0.85, 0.87), respectively. For revascularization, the initially high cAUC declined after the first year but achieved its peak values in long-term observation – mean cAUC in the fifth year of follow-up was 0.84 (95% CI: 0.82, 0.85). While the perfusion abnormality measure maintains high cAUC over a short-term observation for the prediction of ACS, revascularization or any event, the cAUC decreases over time faster than of our model. The model has superior performance to the perfusion abnormality for death prediction at all time points for cAUC as shown in Table 3 (in the internal testing set) and in Table 4 (in the external testing set).

**Table 3:** Comparison of performance in the internal set (*n* = 20,401).

Event and time point	AUC Full model (1)	AUC Perfusion abnormality (2)	AUC Clinical-only model (3)	*p* value (1) versus (2)	*p* value (1) versus (3)	*p* value (2) versus (3)
MACE at 1 year	0.78 (0.77, 0.79)	0.76 (0.75, 0.77)	0.69 (0.68, 0.71)	0.089	**<0.001**	**<0.001**
MACE at 3 years	0.76 (0.75, 0.77)	0.72 (0.71, 0.73)	0.72 (0.71, 0.73)	**<0.001**	**<0.001**	0.788
MACE at 5 years	0.76 (0.75, 0.77)	0.70 (0.69, 0.71)	0.73 (0.72, 0.74)	**<0.001**	**<0.001**	**<0.001**
Death at 1 year	0.80 (0.77, 0.82)	0.61 (0.58, 0.64)	0.76 (0.74, 0.79)	**<0.001**	**0.002**	**<0.001**
Death at 3 years	0.78 (0.76, 0.80)	0.62 (0.60, 0.64)	0.76 (0.74, 0.77)	**<0.001**	<**0.001**	**<0.001**
Death at 5 years	0.77 (0.76, 0.79)	0.61 (0.60, 0.63)	0.75 (0.74, 0.76)	**<0.001**	**<0.001**	**<0.001**
ACS at 1 year	0.73 (0.70, 0.76)	0.73 (0.69, 0.76)	0.70 (0.67, 0.73)	0.972	**0.025**	0.188
ACS at 3 years	0.70 (0.68, 0.72)	0.68 (0.66, 0.71)	0.68 (0.66, 0.70)	0.253	0.054	0.970
ACS at 5 years	0.70 (0.68, 0.71)	0.67 (0.65, 0.69)	0.68 (0.66, 0.69)	0.054	**0.025**	0.621
Revasc. at 1 year	0.79 (0.77, 0.80)	0.81 (0.80, 0.83)	0.70 (0.68, 0.71)	**0.018**	**<0.001**	**<0.001**
Revasc. at 3 years	0.78 (0.76, 0.79)	0.77 (0.75, 0.78)	0.71 (0.70, 0.73)	0.489	**<0.001**	**<0.001**
Revasc. at 5 years	0.77 (0.76, 0.78)	0.75 (0.73, 0.76)	0.71 (0.70, 0.73)	**0.011**	<**0.001**	**<0.001**

All *p* values were obtained using DeLong’s test. The bold values represent statistically significant (*p* < 0.05) comparisons. Abbreviations: *ACS* acute coronary syndrome, *AUC* area under the receiver-operating curve, *MACE* major adverse cardiovascular event, *Revasc*. revascularization.

**Table 4:** Comparison of performance in the external testing set (*n* = 13,988).

Event and time point	AUC Full model (1)	AUC Perfusion abnormality (2)	AUC Clinical-only model (3)	*p* value (1) versus (2)	*p* value (1) versus (3)	*p* value (2) versus (3)
MACE at 1 year	0.74 (0.73, 0.76)	0.71 (0.70, 0.73)	0.68 (0.66, 0.69)	**<0.001**	**<0.001**	**0.001**
MACE at 3 years	0.73 (0.72, 0.74)	0.69 (0.67, 0.70)	0.69 (0.68, 0.71)	**<0.001**	**<0.001**	0.341
Death at 1 year	0.77 (0.74, 0.80)	0.61 (0.58, 0.65)	0.75 (0.72, 0.78)	**<0.001**	0.320	**<0.001**
Death at 3 years	0.76 (0.73, 0.78)	0.61 (0.58, 0.64)	0.72 (0.70, 0.74)	**<0.001**	**0.026**	**<0.001**
ACS at 1 year	0.74 (0.70, 0.78)	0.69 (0.64, 0.74)	0.72 (0.68, 0.76)	**0.021**	0.426	0.343
ACS at 3 years	0.72 (0.69, 0.74)	0.65 (0.62, 0.68)	0.70 (0.68, 0.73)	**<0.001**	0.469	**0.012**
Revasc. at 1 year	0.74 (0.73, 0.76)	0.74 (0.72, 0.76)	0.66 (0.64, 0.68)	0.763	**<0.001**	**<0.001**
Revasc. at 3 years	0.72 (0.70, 0.74)	0.72 (0.70, 0.74)	0.67 (0.65, 0.69)	0.737	**<0.001**	**<0.001**

All *p* values were obtained using DeLong’s test. The bold values represent statistically significant (*p* < 0.05) comparisons. Abbreviations: *ACS* acute coronary syndrome, *AUC* area under the receiver-operating curve, *MACE* major adverse cardiovascular event, *Revasc*. revascularization.

Time-dependent concordances for the prediction of each separate event are shown in Supplementary Table 1. The concordances were higher for all-cause death than for ACS, or revascularization.

### External testing

**Fig. 3:**
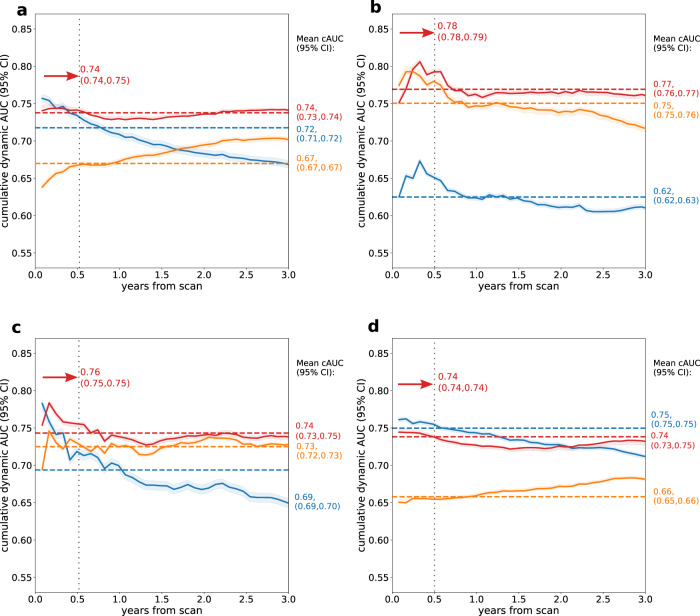
Cumulative dynamic areas under the receiver operating characteristic curve (cAUC) in the external testing set. The cAUCs are presented for the prediction of any adverse event (**a**) as well as specific events: all-cause death (**b**), acute coronary syndrome (**c**), and revascularization (**d**) in the external dataset (*N* = 13,988). The red, blue, and orange lines represent cAUC for the time-to-event model, perfusion abnormality, and clinical-only model, respectively. The red, blue, and orange dashed lines represent mean cAUC for the time-to-event model, perfusion abnormality, and clinical-only model, respectively. Shaded areas represent 95% confidence intervals (CI). The red arrows show the cAUC values of time-to-event deep learning averaged over the first six months after the scan (vertical dashed line).

Similar to internal testing, the model preserved relatively constant cAUC within 3 years from the baseline scan, while for perfusion abnormality, the cAUC declined gradually over time (Fig. 3). For each of the events, the best performance was observed for short-time prediction – in the first six months after scan mean cAUC for ACS and all-cause death reached 0.76 (95% confidence interval [CI]: 0.75, 0.76) and 0.78 (95% CI: 0.78, 0.79), respectively. Our model outperforms perfusion abnormality at all time points for the prediction of death. Time-dependent concordance for the prediction of each event in the external set is presented in Supplementary Table 1.

The time-to-event model outperformed both perfusion abnormality and the clinical-only model in the external testing set at 1 and 3 years from scan. The area under the receiver operating curve (AUC) for any MACE at 1 year from the scan was 0.74 (95% CI: 0.73,0.76) for the time-to-event model, 0.71 (95% CI: 0.70,0.73) for perfusion abnormality, and 0.68 (95% CI: 0.66,0.69) for the clinical-only model. At 3 years from scan, the AUC for any MACE was 0.73 (95% CI: 0.72,0.74) for the time-to-event model, 0.69 (95% CI: 0.67,0.70) for perfusion abnormality, and 0.69 (95% CI: 0.68,0.71) for the clinical-only model. The time-to-event model performed better than perfusion abnormality at 1 and 3 years from scan in the prediction of death or ACS and better than the clinical-only model in the prediction of revascularization. Detailed comparison of AUC values for all events for the time-to-event model compared with perfusion abnormality and the clinical-only model is shown in Table 3. Receiver-operating curve (ROC) plots for the prediction of death, ACS, and revascularization at 1, 3 and 5 years in the external testing sets using time-to-event model are shown on Supplementary Fig. 1. Sensitivity analysis showed no significant effect on the prediction of other events or composite MACE outcome, but it decreased performance for prediction of revascularization when revascularization events within 180 days from the scan are removed (Supplementary Table 2). The time-to-event model also outperformed the multivariable Cox regression model in the prediction of all types of events (Supplementary Table 3).

### Individual prediction and explanation

**Fig. 4:**
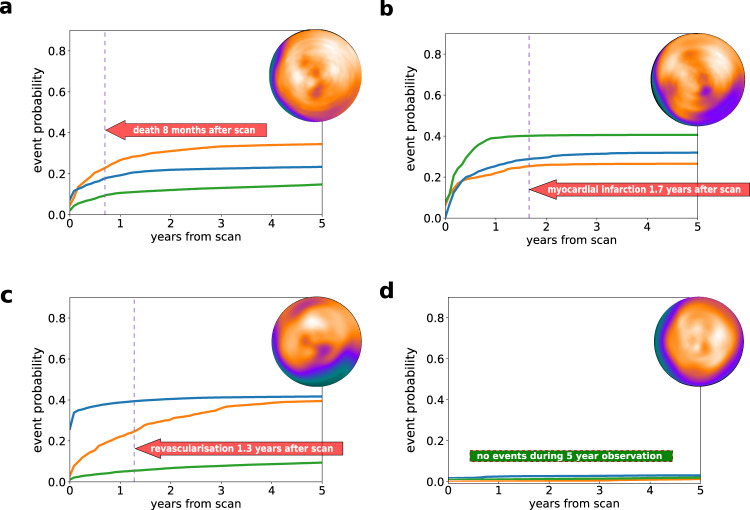
Patient-level prediction of time-dependent risk of major adverse cardiovascular events. Individual prediction of event probability in 4 different patients: (**a**) A 58-year-old male with no history of CAD, stress TPD of 2%, and diabetes; (**b**) A 76-year-old male with history of PCI and CABG, stress TPD of 3%, and family history of CAD and dyslipidemia; (**c**) A 63-year-old male with no history of CAD, stress TPD of 20%, and no other risk factors; (**d**) A 60-year-old male with no history of CAD, stress TPD of 1% and no risk factors. Curves represent predicted cumulative event probability of death (orange lines), acute coronary syndrome (green lines), and revascularization (blue lines) as a function of time. The vertical dashed lines mark the time of the true event. CABG – coronary artery bypass grafting, CAD coronary artery disease, PCI percutaneous coronary intervention, TPD total perfusion deficit (measure of perfusion abnormality).

**Fig. 5:**
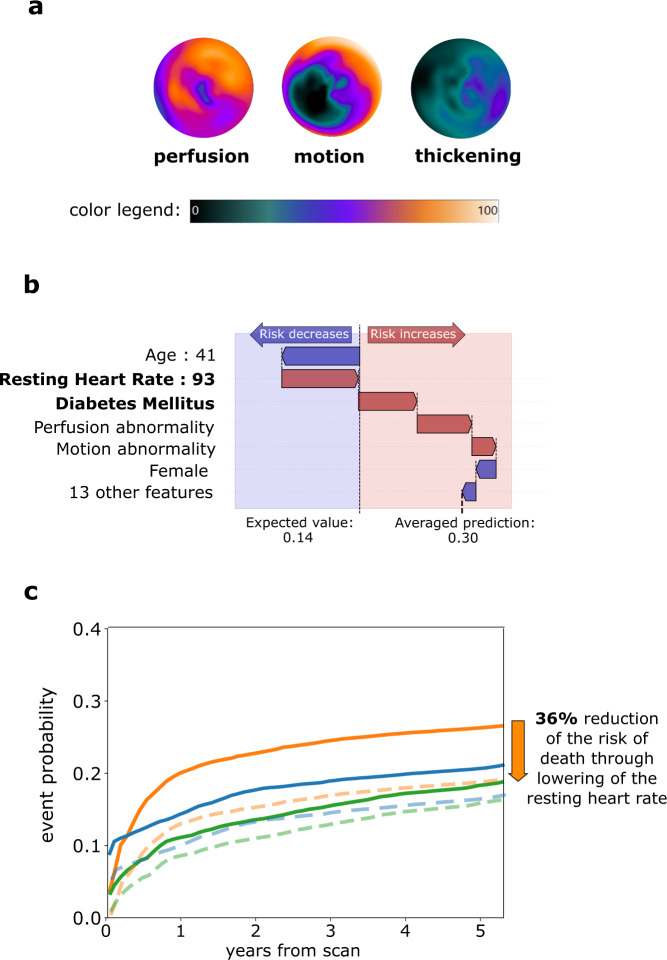
Patient-level risk explanation with simulated modification of risk factors. **a** Polar map visualization of perfusion, motion, and thickening of a 41-year-old female with no history of CAD, LVEF of 39%, and BMI of 31. Volume and degree of affected myocardium (0-100%) is visualized using color on the polar map, as shown on a color legend. **b** Waterfall plots present the influence of top features (including averaged contributions from polar maps) on prediction of average risk of death. The plot starts at the top at the mean population probability (expected value, dotted vertical line) and ends at the bottom with the final individual probability. Red arrows pointing to the right indicate that the feature increases the risk of death, while blue arrows pointing to the left indicate that the feature decreases the risk. Patient features that point to potential actionable interventions through medication or lifestyle modification are highlighted using **bold font**. **c** Simulated modification of risk factors through reduction of resting heart rate to 70/min reduces the average risk of death by 36%. The predicted probabilities of death, ACS and revascularization before the intervention is shown as orange, green and blue lines, respectively. The predicted probabilities of death, ACS and revascularization after heart rate reduction are shown as orange, green and blue dashed lines, respectively. ACS acute coronary syndrome, CAD coronary artery disease, LVEF left ventricular ejection fraction, BMI body mass index, PCI percutaneous coronary intervention, CABG coronary artery bypass grafting.

Examples of individualized predictions for four patients who experienced different types of outcomes in the follow-up period are shown in Fig. 4. The prediction of our model is presented as three cumulative incidence functions – separately for each type of event. The individual prediction plot is accompanied by a waterfall plot (Fig. 5) – providing an explanation of the highest predicted risk that highlights how the polar maps and clinical features contribute to the overall risk. The waterfall plot allows for visualization of both the extent of influence (length of the arrow) and direction (increasing risk of the event – red arrow pointing to the right, decreasing risk – blue arrow pointing to the left). In the presented case, a 41-year-old female with heart failure with moderately reduced ejection fraction and moderate perfusion deficits is identified as having a high risk of death. Explanation of the prediction shows the elevated resting heart rate as one of the factors having the greatest contribution to the elevated risk. Simulating the reduction of the resting heart rate to 70/min shows that optimal guideline-guided management could lower average predicted risk of death by 36%. Inference using our model took below 12 milliseconds per patient case on an Apple MacBook Pro laptop computer.

## Discussion

Leveraging a large cardiac imaging registry, we developed a deep learning approach for individual risk computation that allows time-dependent and event-specific predictions jointly from clinical and cardiac imaging data. We obtain time- and event-specific risk estimation and provide visually intuitive graphs for individual risk explanations. The model provides risk estimates over-time, for all-cause death, ACS, and late revascularization separately, with easy-to-understand patient-level explanations. We evaluated our model in a large, multi-site external dataset as well as with internal 10-fold cross-validation. Good performance in the external testing set points to the ability of our model to generalize to unseen real-life data from new centers. The model relies on the combined predictive potential of the clinical features, stress test data, and direct image analysis, similarly to the way clinicians try to integrate all available information to provide the most accurate study interpretation. Moreover, this approach also leverages time-to-event data to provide more robust risk estimation over time, which could potentially be applied to a broad range of AI tasks.

Previous prognostic studies estimated risk jointly using composite adverse events and without the use of time-to-event data^5^. A high-risk of death in the next year is a very different scenario than a risk of hospital admission or revascularization over 10 years – but our current presentation and assessment of data lack this granularity. Recently, non-linear AI survival models have demonstrated practical implementations in healthcare^7–9^ with state-of-the-art performances that are comparable to or improve the performance of traditional Cox proportional hazard models^10^. Examples of such models include precision genomic prognostication in patients receiving cancer treatment^11^, prediction of oral cancer survival^12^ as well as of progression of potentially malignant disorders to cancer^13^. A large scale, multisite study investigated the use of deep neural networks trained using full electronic health records data in prediction multiple medical events^14^. This is however, to our knowledge, the first study to evaluate prediction at multiple time points of multiple events in a large multi-site registry of cardiovascular imaging data that also explicitly takes advantage of time-to-event data during model training.

Patient level-explanation may be crucial for the clinical adoption of AI in medical imaging^15,16^, but this approach was not previously applied as a joint explanation of direct imaging data and clinical variables for individualized risks of specific events. Such explanations may point to abnormalities in the imaging data as well as to some clinical features that drive the increased risk for a given adverse event, potentially allowing for a more comprehensive assessment of patient’s condition. Event-specific predictions within a single model can be presented at the time of review of imaging and may enable physicians to practice precision medicine, with individually tailored treatments and preventive measures. For instance, a prediction of high risk of all-cause death could encourage more frequent follow-up visits and additional diagnostic tests, while a high risk of ACS and revascularization could indicate that the patient is a candidate for revascularization or needs intensification of medical therapy.

In addition to informing the physician about the rationale behind model predictions, the visualization of factors contributing to increased risk of adverse events might serve as a powerful tool in shared decision-making after the exam, utilizing all available information^17^. When discussed with the patient, a special focus might be given to modifiable risk factors such as high BMI^18^, hypertension^19^, diabetes, and dyslipidemia^20^, leading to optimal, goal-directed medical therapy of these risk factors. That could be a starting point for a discussion on how these factors can be targeted through lifestyle modifications and medications. Such an approach could be an important step towards patient empowerment and could improve adherence to physicians’ recommendations. However, it is important to acknowledge the limitations of SHapley Additive exPlanations (SHAP) - derived feature importance^21^, especially that they do not imply casual relations between the input features and the outcome. For this reason, the waterfall plots (Fig. 5) that were generated based on SHAP values should be considered an illustrative tool and should be interpreted with caution.

Interestingly, in the external testing set, we found that the perfusion abnormality variable had lower performance than the clinical-only time-to-event model in predicting all-cause mortality at any time point. This confirms that clinical features, such as age and medical comorbidities, are important determinants of all-cause mortality and have previously been shown to influence the “warranty period” of normal perfusion on MPI^22^. Additionally, myocardial perfusion may change in response to anti-anginal therapies and thus would not be expected to be an accurate predictor of long-term hard outcomes^23^. However, unsurprisingly the revascularization prediction performance was similar for the perfusion abnormality and the full time-to-event model and higher than for the clinical-only time-to-event model. This is expected because physicians may rely on perfusion information when making revascularization decisions^24,25^, which could lead to overestimation of its prediction performance for the revascularization.

Our study has several limitations. First, we have only assessed all-cause mortality and could, therefore, not differentiate between cardiac and non-cardiac deaths. We separately considered the major cardiovascular events of ACS and revascularization. However, other possible events like atrial fibrillation, worsening of heart failure, or sub-classification of ACS (presence of ST-elevation) were not available for analysis due to the multi-site, retrospective nature of the imaging registry. In our model, the risk for death and ACS is estimated independently from the risk of revascularization, thus allowing for the event-specific assessment of patient’s prognosis, but it should be noted that an increased risk of ACS and revascularization would lead physicians to consider the same preventive strategies. Furthermore, while the dynamic cAUC of the standard quantitative perfusion analysis decreases over time for the revascularization prediction, our model maintains higher cAUC for the revascularization prediction in the long term. The performance of our model could be further improved by utilizing data from other imaging modalities^26^. For instance, computed tomography attenuation correction scans could be used to automatically calculate calcium score, which could be included in the time-to event model^27^. Finally, the usefulness of the time-to-event predictions has not been evaluated in prospective studies. This is understandable given the novelty of the proposed methods. Further investigation is needed to assess if the additional temporal dimension of the model’s prediction and its ability to differentiate the risk of specific events can improve physicians’ workflows and lead to better clinical decisions.

The proposed deep learning model, using cardiac perfusion images and clinical data with time-to-event specific outcomes, provides a robust prediction of the risk of all-cause death, ACS, and revascularization. The model significantly improved the prediction of all-cause death and the composite MACE outcome, while also improving the prediction of ACS in the external testing population. By presenting the individualized patient-specific post-scan risk assessment over time in an intuitive manner for the clinicians and patients, our approach can potentially help better address patient risk and guide management that is tailored to the patient’s individual risk profile.

## Methods

### Patient populations

For the training and internal validation, we included 20,418 scans from five international centers participating in the prospective, multi-site Registry of Fast Myocardial Perfusion Imaging with Next generation SPECT (REFINE SPECT)^28^. We included all consecutive patients who underwent clinically indicated SPECT MPI from 2009 to 2014. We excluded 17 patients without gated studies, leaving a total of 20,401 patients.

### Definition of events

Patients were followed for MACE, which was defined as all-cause death, myocardial infarction, unstable angina, and revascularization (surgical or percutaneous). Non-fatal myocardial infarction was defined as hospitalization for cardiac chest pain or anginal equivalent with positive cardiac biomarkers^29^. Unstable angina was defined as recent onset or escalating cardiac chest pain with negative cardiac biomarkers. All outcomes were adjudicated by experienced cardiologists after considering all available clinical data. We chose three outcomes as events of interest: death, acute coronary syndrome (ACS) - defined as either non-fatal myocardial infarction or admission for unstable angina, and revascularization (with percutaneous coronary intervention or coronary artery bypass grafting). For each patient, only the first occurring event was considered and therefore, each patient had either one of the three events or no events. If a patient presented with ACS and had revascularization on the same day, that event was considered as an ACS. If a patient had either ACS or revascularization and died on the same day, that event was considered as a death. For area under receiver-operating curve (AUC) analysis, events that occurred up to a given time-point were considered as positive events, and if the event occurred after the specified time point, the patient was considered event-free.

### External cohort

The external testing population included an additional 13,988 patients who underwent clinically indicated SPECT MPI with MACE follow–up at a separate three external centers: Oklahoma Heart Hospital (*n* = 6034), University of Calgary Hospital (*n* = 2985) and Yale New Haven Hospital (*n* = 4969). All outcomes were adjudicated using the same criteria as in the training cohort.

### Image collection

Patients were imaged with either a DSPECT (Spectrum-Dynamics, Caesarea, Israel), GE Discovery NM 530c, or NM/CT570c (GE Healthcare, Haifa, Israel) camera system. Patients underwent either symptom-limited exercise testing or pharmacologic stress. Additional details regarding imaging protocols and acquisition have been previously described^28^.

After anonymization, all images were transferred to Cedars-Sinai Medical Center, where quality control was performed by experienced core laboratory technologists without knowledge of the clinical data. Left ventricular (LV) myocardial contours were computed and verified by an experienced nuclear medicine technologist using standard clinical software^30^. Polar maps of the LV, representing compressed form of images, were automatically generated from the images. Five polar maps were derived for each patient including perfusion, motion, thickening, cardiac phase, and amplitude. Clinical data and images from the external centers were de-identified and transferred to Cedars-Sinai. This study complies with the Declaration of Helsinki.

### Ethical approvals

The institutional review boards at Cedars-Sinai and the participating sites approved the collection of data for the registry: Cedars-Sinai Institutional Review Board, PeaceHealth System Institutional Review Board (Oregon Heart and Vascular Institute), Ottawa Health Science Network Research Ethics Board, Partners Human Research Committee (Brigham and Women’s Hospital), Assuta Medical Centers Ethics Committee, Western Institutional Review Board (Oklahoma Heart Hospital Research Foundation), Conjoint Health Research Ethics Board of the University of Calgary, and Yale University Institutional Review Board. Informed, written consent was obtained from the subjects (or their legally authorized representative) in Cedars Sinai Medical Center and Brigham and Women’s Hospital. In the remaining sites, waiver of consent was granted by the respective local institutional review boards.

### Clinical features

Clinical and stress test results were collected according to the protocol of the REFINE SPECT registry. All the clinical features used in the model are listed in Supplementary Table 4.

### Design and training of the event-specific deep learning network

We employed a deep-learning-based approach capable of learning the distribution of event ‘hitting times’ directly from data. We extended the DeepHit architecture and associated loss function^31^ and implemented with the PyTorch framework^32^. To allow the network to process images, we added convolutional layers capable of directly interrogating perfusion, motion, wall thickening, and phase polar maps and combining the imaging data with 15 clinical features that were chosen based on our previous work on the minimum set of variables for machine learning cardiovascular event prediction^33^. The network consists of two main parts:

1. convolutional part that processes the 28 x 36 x 5 input of 5 normalized polar maps consisting of 2 convolution blocks, each with 3×3 convolution kernels, batch normalization, dropout, and Leaky Rectified Linear Unit (ReLU) layers, which were added to prevent overfitting.
2. clinical features are added in the fully connected layer, with 512 nodes and 15 clinical features passed to a separate fully connected layer with 32 nodes. The output of these layers is concatenated and passed to the DeepHit network described by Lee et al.^31^, with 256 nodes in a single shared layer and 256 nodes in each of the three event-specific layers.

The output of the model is a 2-D 3 × 131 array of shape, representing probabilities of each of the events occurring at time 0 and every 30 days up to the maximum follow up time. We used loss functions proposed by Lee et al^31^. with modification by Kvamme et al^32^.

Missing values (Supplementary Table 5) were imputed using mean or mode (in case of categorical features) values in the training set. This method was previously shown to perform similar to other data imputation techniques^34^.

The important aspect of the architecture is the ability to handle multiple competing events^35^ and generate predictions separately for each of them. The resulting architecture is shown in Supplementary Fig. 2. The model generated predictions in the form of a 2-D array of monthly event probabilities for multiple events.

### Additional analyses

To evaluate the usefulness of combining clinical and imaging data in a model, we trained and tested a separate model that used clinical features only. This model utilizes the same architecture as the time-to-event model, but without image input. Additionally, we performed a sensitivity analysis in the external testing population to investigate the effect of removing cases with revascularization events within 180 days from the MPI. We compared the AUC for the prediction of revascularization, death, ACS, and MACE in the external dataset at 1 year and 3 years from scan with and without removing the early revascularization cases.

### Comparison with Cox regression model

For comparison, we created a multivariable Cox regression model that used all clinical features utilized by the time-to-event model and stress total perfusion deficit (perfusion abnormality). This model was trained in the internal set and evaluated in the external testing set. We compared AUC for the prediction of death, ACS, revascularization, and MACE at 1 and 3 years from scan.

### Internal Training and testing routine

The model was trained and tested in a 10-fold repeated hold-out regimen. The development set was randomly divided into 10 samples (folds) with the same fraction of each MACE event (stratified split). Then, 10 separate models were trained, each using 9 of 10 folds for training and the remaining one for testing. Within the 90% of training data, the model randomly selected 20% of cases that were used for model hyperparameter optimization in this fold. There was no overlap of training data with the testing data at any point. Testing results from each of the 10 folds and 10 models were concatenated for robust assessment of the overall performance in unseen data.

### External testing

The generalizability of the approach to data from new medical centers was evaluated in external testing regimen. For the robust estimation of external performance, each of the 10 models generated in 10-fold cross-validation of REFINE SPECT cohort was evaluated in a separate external cohort from new three new centers. Performance was then evaluated separately for the 10 sets of predictions and presented as mean with 95% confidence intervals after bootstrapping.

### Patient-specific explanations

We provide explanations of individualized predictions made by the algorithm. This approach allows for the identification of important patient-specific features driving the prediction and provides a feature importance ranking for each patient, separately for each of the three separate outcomes. The individual explanation of the predicted probability of each of the target events was achieved through the generation of SHapley Additive exPlanations (SHAP values)^36^. To provide a meaningful explanation, we summed the SHAP values for all pixels in each image input and presented them alongside the importance of clinical features in the form of waterfall plots. For each of the top contributing features, the water fall plot visualizes how strongly the given feature increases or decreases the risk for the specific events in a specific patient.

### Statistical analysis

Continuous variables were expressed as median and (interquartile ranges [IQR]). Two-sided Kruskal-Wallis test were used to compare differences in median values. Categorical variables were compared using Fisher’s exact test. A *p* value <0.05 was considered statistically significant. Univariable comparisons and summary statistics were computed using R 4.1.2 and R Studio. Details on used software packages and versions are given in the Supplementary Table 6.

The predictive performance of the model was evaluated using time-dependent concordance index^37^ that extends the concordance index for time-dependent predictions, and cumulative dynamic area under the receiver-operating characteristic (ROC) curve (cAUC)^38^ as implemented in the scikit-survival python package. This measure reflects the probability that, given two randomly chosen patients, one having failed before time <T > and the other having failed after <T > , the prognostic marker will be correctly ranked. We used plots of the cAUC values as a function of time from scan to visualize the ability to capture temporal changes in the risk of adverse events. The 95% confidence limits for the cAUC curves were established using bootstrapping (100 samples with replacement). We compared the cAUC of our model’s output with the clinical-only model and with the extent of perfusion abnormality (stress total perfusion deficit)^30^, which is an established, clinically used quantitative MPI variable. In previous studies, total perfusion deficit measure demonstrated efficient risk stratification^39^ and identification of patients who may benefit from early revascularization^24^.

Additionally, we evaluated the model using area under the ROC curves (AUC) at 3-time points in the internal testing set (1, 3, and 5 years from scan), separately for all-cause death, ACS, and revascularization at each time point and for each event using DeLong’s test.

### Compliance with recommendations for machine-learning-related research

This study was designed and conducted following the transparent reporting of a multivariable prediction model for individual prognosis or diagnosis (TRIPOD)^40^ checklist that is included as Supplementary Table 7.

### Reporting summary

Further information on research design is available in the Nature Research Reporting Summary linked to this article.

## Supplementary information

Supplementary Material

REPORTING SUMMARY

## Data Availability

De-identified data supporting this study may be shared based on reasonable written request to the corresponding author. Access to de-identified data will require a Data Access Agreement and IRB clearance, which will be considered by the institutions who provideded the data for this research.

## Code availability

The source code will be shared using a Creative Commons NC-ND 4.0 international license upon reasonable written request to the corresponding author and requires a research use agreement. The list of software packages for the deep learning model and for statistical analyses and their versions is provided in Supplementary Table 6.
