# Supplementary material for: Time and event-specific deep learning for personalized risk assessment after cardiac perfusion imaging
Source: NPJ Digit Med. 2023 May 1;6:78. doi: 10.1038/s41746-023-00806-x (PMC10151323; doi:10.1038/s41746-023-00806-x)
Supplement: Supplementary file 1 — Supplementary Material [file 41746_2023_806_MOESM1_ESM.docx]

**Supplementary File**

Time and event-specific deep learning for personalized risk assessment after cardiac perfusion imaging

Konrad Pieszko^1,2^, Aakash D. Shanbhag^1^, Ananya Singh^1^, M. Timothy Hauser^3^, Robert J. H. Miller^1,4^, Joanna X. Liang^1^, Manish Motwani^5,6^, Jacek Kwieciński^1,7^, Tali Sharir^8^, Andrew J. Einstein^9^, Mathews B. Fish^10^, Terrence D. Ruddy^11^, Philipp A. Kaufmann^12^, Albert J. Sinusas^13^, Edward J. Miller^13^, Timothy M. Bateman^14^, Sharmila Dorbala^15^, Marcelo Di Carli^15^, Daniel S. Berman^1^, Damini Dey^1^, Piotr J. Slomka^1^

1) Departments of Medicine (Division of Artificial Intelligence in Medicine), Imaging, and Biomedical Sciences, Cedars-Sinai Medical Center, Los Angeles, CA, USA

2) Department of Interventional Cardiology and Cardiac Surgery, Collegium Medicum, University of Zielona Góra, Zielona Góra, Poland

3) Department of Nuclear Cardiology, Oklahoma Heart Hospital, Oklahoma City, Oklahoma

4) Department of Cardiac Sciences, University of Calgary and Libin Cardiovascular Institute, Calgary, Alberta, Canada

5) Institute of Cardiovascular Science, University of Manchester, Manchester, UK

6) Department of Cardiology, Manchester Heart Institute, Manchester Royal Infirmary, Manchester University NHS Foundation Trust, UK

7) Department of Interventional Cardiology and Angiology, Institute of Cardiology, Warsaw, Poland

8) Department of Nuclear Cardiology, Assuta Medical Centers, Tel Aviv, Israel

9) Division of Cardiology, Department of Medicine and Department of Radiology, Columbia University Irving Medical Center and NewYork-Presbyterian Hospital, New York, New York

10) Oregon Heart and Vascular Institute, Sacred Heart Medical Center, Springfield, OR, USA

11) Division of Cardiology, University of Ottawa Heart Institute, Ottawa, ON, Canada

12) Department of Nuclear Medicine, Cardiac Imaging, University Hospital Zurich, Zurich, Switzerland

13) Section of Cardiovascular Medicine, Department of Internal Medicine, Yale University School of Medicine, New Haven, CT, USA

14) Cardiovascular Imaging Technologies LLC, Kansas City, MO, USA

15) Department of Radiology, Division of Nuclear Medicine and Molecular Imaging, Brigham and Women's Hospital, Boston, MA, USA

**Corresponding Author**

Piotr J. Slomka, PhD

Department of Medicine (Division of Artificial Intelligence) Cedars-Sinai Medical Center

8700 Beverly Blvd, Suite Metro 203

Los Angeles, CA 90048

Email: Piotr.Slomka@cshs.org

## Supplementary Table 1

Time-dependent concordance in internal and external testing sets (n=13988)

|  |  | **Death** | **Acute coronary syndrome** | **Revascularization** |
| --- | --- | --- | --- | --- |
| Mean time-dependent concordance index (95% CI) | 10-fold hold out testing | 0.79  (0.77, 0.80) | 0.74  (0.71, 0.75) | 0.77  (0.75, 0.78) |
|  | External testing | 0.73 (0.72, 0.73) | 0.75 (0.74, 0.75) | 0.69 (0.69, 0.7) |

Time-dependent concordance reflects the probability that, given two randomly chosen patients, one having failed before time T and the other having failed after T, the prognostic marker will be correctly ranked; CI – confidence interval.

##

## Supplementary Table 2

Sensitivity analysis in the external testing set (n=13988) for removal of cases with revascularization events within 180 days from scan in the external testing set

| Event | Years from scan | Area under the receiver-operating curve | | p-value for DeLong’s test |
| --- | --- | --- | --- | --- |
|  |  | Including cases with revascularization events (n=13,988) | Cases with revascularization events removed (n=13,379) |  |
| MACE | 1 | 0.74 (0.73,0.76) | 0.73 (0.71,0.75) | 0.513 |
|  | 3 | 0.73 (0.72,0.74) | 0.72 (0.71,0.74) | 0.287 |
| Death | 1 | 0.77 (0.74,0.80) | 0.78 (0.75,0.80) | 0.822 |
|  | 3 | 0.76 (0.73,0.78) | 0.76 (0.74,0.78) | 0.723 |
| ACS | 1 | 0.74 (0.70,0.78) | 0.75 (0.71,0.79) | 0.745 |
|  | 3 | 0.72 (0.69,0.74) | 0.73 (0.70,0.75) | 0.625 |
| Revasc. | 1 | 0.74 (0.73,0.76) | 0.62 (0.55,0.68) | <0.001 |
|  | 3 | 0.72 (0.70,0.74) | 0.65 (0.61,0.68) | <0.001 |

Revasc.- revascularization;

## Supplementary Table 3

Comparison with multivariable Cox regression model in the external testing set

| Event | Years from scan | Area under the receiver-operating curve | | p-value for DeLong’s test |
| --- | --- | --- | --- | --- |
|  |  | Time-to-event deep learning model | Cox regression model |  |
| MACE | 1 | 0.74 (0.73,0.76) | 0.70 (0.69,0.72) | <0.001 |
|  | 3 | 0.73 (0.72,0.74) | 0.71 (0.70,0.72) | <0.001 |
| Death | 1 | 0.77 (0.74,0.80) | 0.75 (0.72,0.78) | 0.008 |
|  | 3 | 0.76 (0.73,0.78) | 0.74 (0.72,0.77) | 0.017 |
| ACS | 1 | 0.74 (0.70,0.78) | 0.72 (0.68,0.77) | 0.05 |
|  | 3 | 0.72 (0.69,0.74) | 0.69 (0.66,0.72) | 0.015 |
| Revasc. | 1 | 0.74 (0.73,0.76) | 0.67 (0.65,0.69) | <0.001 |
|  | 3 | 0.72 (0.70,0.74) | 0.67 (0.65,0.68) | <0.001 |

Revasc.- revascularization;

## Supplementary Table 4

Input features for the models

|  | Imaging data | Clinical features | Stress-test results |
| --- | --- | --- | --- |
| Full model | 5 polar maps (28x36 pixels)   1. Stress perfusion 2. Motion 3. Thickening 4. Phase angle 5. Phase amplitude | Age  Gender  Body mass index  History of PCI  History of TAVR  History of CABG  History of cardiac transplant  Hypertension  Diabetes mellitus  Dyslipidemia  Family history  Resting heart rate | Stress test type  Stress heart rate peak  Stress systolic BP peak |
| Clinical-only model |  | Age  Gender  Body mass index  History of PCI  History of TAVR  History of CABG  History of cardiac transplant  Hypertension  Diabetes mellitus  Dyslipidemia  Family history  Resting heart rate | Stress test type  Stress heart rate peak  Stress systolic BP peak |

## Supplementary Table 5

Missing values

| Variable name | Fraction of missing values in the internal set  (n=20401), % | Fraction of missing values in the external set (n=13988) | | | |
| --- | --- | --- | --- | --- | --- |
|  |  | Site 1 (n=6034) | Site 2  (n=4969) | Site 3 (n=2985) | Total, % |
| Polar maps | 0% | 0 | 0 | 0 | 0% |
| Age | 0% | 0 | 0 | 0 | 0% |
| Gender | 0% | 0 | 0 | 0 | 0% |
| Body mass index | 0.0007% | 3 | 0 | 213 | 2% |
| History of PCI | 0.0002% | 0 | 0 | 0 | 0% |
| History of TAVR | 0.0002% | 0 | 0 | 0 | 0% |
| History of CABG | 0.0002% | 0 | 0 | 0 | 0% |
| History of cardiac transplant | 0.0002% | 0 | 0 | 0 | 0% |
| Hypertension | 0.0002% | 0 | 0 | 0 | 0% |
| Diabetes mellitus | 0.0002% | 0 | 0 | 0 | 0% |
| Dyslipidemia | 0.0002% | 0 | 0 | 0 | 0% |
| Family history | 0.0002% | 0 | 0 | 0 | 0% |
| Resting heart rate | 0.0008% | 6 | 32 | 679 | 5.1% |
| Stress test type | 0.0002% | 0 | 0 | 0 | 0% |
| Stress heart rate peak | 0.002% | 7 | 125 | 803 | 6.7% |
| Stress systolic BP peak | 0.002% | 7 | 36 | 835 | 6.2% |

## Supplementary Table 6

Software packages used in data analysis with versions

| **Name** | **Version** | **Additional details** |
| --- | --- | --- |
| Python | 3.8.12 |  |
| pytorch | 1.10.0 | Deep learning framework |
| scikit-learn | 0.24.2 | General machine learning framework |
| pycox | 0.2.1 | Implementation of DeepHit model loss functions, time-dependent concordance computation |
| pandas | 1.2.4 | Data manipulation |
| sklearn-pandas | 2.2.0 | Data preprocessing |
| shap | 0.40.0 | SHAP values computation and plotting |
| matplotlib | 3.1.1 | Plotting |
| scikit-survival | 0.16.0 | Cumulative area under receiver operating curve (cAUC) computation |
| R | 4.1.1 |  |
| dplyr | 1.0.7 | Data manipulation |
| gtsummary | 1.4.2 | Table generation, data summary |

## Supplementary Table 7

TRIPOD checklist for prediction model development (D) and validation (V).

| **Section/Topic** | **Item** |  | **Checklist Item** | **Page** |
| --- | --- | --- | --- | --- |
| **Title and abstract** | | | | |
| Title | 1 | D;V | Identify the study as developing and/or validating a multivariable prediction model, the target population, and the outcome to be predicted. | 1 |
| Abstract | 2 | D;V | Provide a summary of objectives, study design, setting, participants, sample size, predictors, outcome, statistical analysis, results, and conclusions. | 2 |
| **Introduction** | | | | |
| Background and objectives | 3a | D;V | Explain the medical context (including whether diagnostic or prognostic) and rationale for developing or validating the multivariable prediction model, including references to existing models. | 3-4 |
|  | 3b | D;V | Specify the objectives, including whether the study describes the development or validation of the model or both. | 4 |
| **Methods** | | | | |
| Source of data | 4a | D;V | Describe the study design or source of data (e.g., randomized trial, cohort, or registry data), separately for the development and validation data sets, if applicable. | 13,14 |
|  | 4b | D;V | Specify the key study dates, including start of accrual; end of accrual; and, if applicable, end of follow-up. | 13 |
| Participants | 5a | D;V | Specify key elements of the study setting (e.g., primary care, secondary care, general population) including number and location of centres. | 13 |
|  | 5b | D;V | Describe eligibility criteria for participants. | 13 |
|  | 5c | D;V | Give details of treatments received, if relevant. | - |
| Outcome | 6a | D;V | Clearly define the outcome that is predicted by the prediction model, including how and when assessed. | 13-14 |
|  | 6b | D;V | Report any actions to blind assessment of the outcome to be predicted. | - |
| Predictors | 7a | D;V | Clearly define all predictors used in developing or validating the multivariable prediction model, including how and when they were measured. | Supplementary Table 4 |
|  | 7b | D;V | Report any actions to blind assessment of predictors for the outcome and other predictors. | - |
| Sample size | 8 | D;V | Explain how the study size was arrived at. | 13 |
| Missing data | 9 | D;V | Describe how missing data were handled (e.g., complete-case analysis, single imputation, multiple imputation) with details of any imputation method. | 16 |
| Statistical analysis methods | 10a | D | Describe how predictors were handled in the analyses. | 15-16 |
|  | 10b | D | Specify type of model, all model-building procedures (including any predictor selection), and method for internal validation. | 15-16 |
|  | 10c | V | For validation, describe how the predictions were calculated. | 17-18 |
|  | 10d | D;V | Specify all measures used to assess model performance and, if relevant, to compare multiple models. | 17-18 |
|  | 10e | V | Describe any model updating (e.g., recalibration) arising from the validation, if done. | - |
| Risk groups | 11 | D;V | Provide details on how risk groups were created, if done. | - |
| Development vs. validation | 12 | V | For validation, identify any differences from the development data in setting, eligibility criteria, outcome, and predictors. | Table:  1,2 |
| **Results** | | | | |
| Participants | 13a | D;V | Describe the flow of participants through the study, including the number of participants with and without the outcome and, if applicable, a summary of the follow-up time. A diagram may be helpful. | p. 12 Table 1,2 |
|  | 13b | D;V | Describe the characteristics of the participants (basic demographics, clinical features, available predictors), including the number of participants with missing data for predictors and outcome. | Table 1 |
|  | 13c | V | For validation, show a comparison with the development data of the distribution of important variables (demographics, predictors and outcome). | Table 2 |
| Model development | 14a | D | Specify the number of participants and outcome events in each analysis. | Table 1,2 |
|  | 14b | D | If done, report the unadjusted association between each candidate predictor and outcome. | Table 1,2 |
| Model specification | 15a | D | Present the full prediction model to allow predictions for individuals (i.e., all regression coefficients, and model intercept or baseline survival at a given time point). | 5-6 |
|  | 15b | D | Explain how to the use the prediction model. | 16 |
| Model performance | 16 | D;V | Report performance measures (with CIs) for the prediction model. | Table 3,4, Fig 2, 3 |
| Model-updating | 17 | V | If done, report the results from any model updating (i.e., model specification, model performance). | - |
| **Discussion** | | | | |
| Limitations | 18 | D;V | Discuss any limitations of the study (such as nonrepresentative sample, few events per predictor, missing data). | 10-11 |
| Interpretation | 19a | V | For validation, discuss the results with reference to performance in the development data, and any other validation data. | 8-10 |
|  | 19b | D;V | Give an overall interpretation of the results, considering objectives, limitations, results from similar studies, and other relevant evidence. | 8-11 |
| Implications | 20 | D;V | Discuss the potential clinical use of the model and implications for future research. | 9 |
| **Other information** | | | | |
| Supplementary information | 21 | D;V | Provide information about the availability of supplementary resources, such as study protocol, Web calculator, and data sets. | 19 |
| Funding | 22 | D;V | Give the source of funding and the role of the funders for the present study. | 19 |

## Supplementary Figure 1


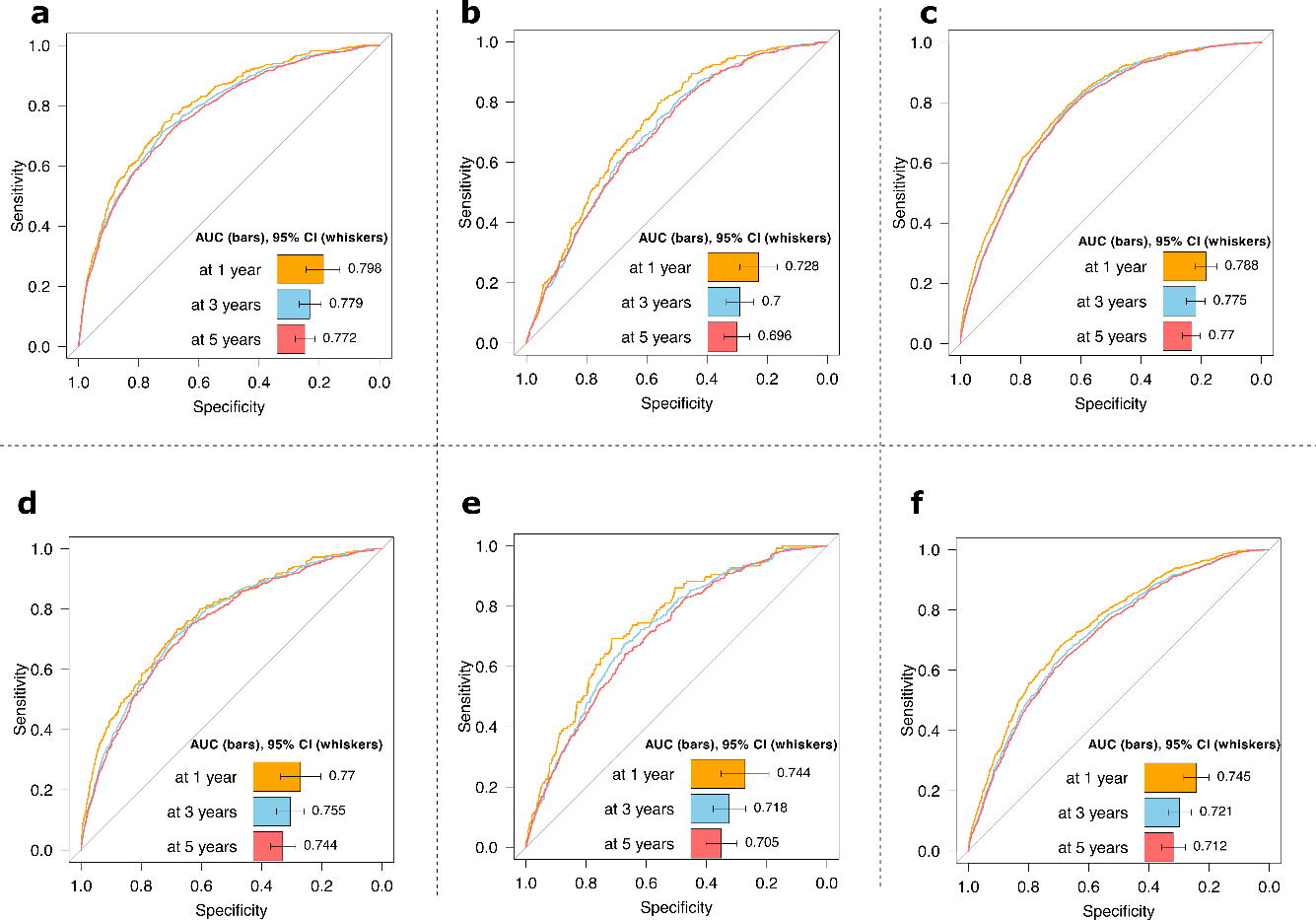


**Receiver operating curves** for prediction of individual adverse cardiac events by proposed model at various time points (1, 3, and 5 years from scan) in the internal testing set (10-fold cross-validation, n=20,401) for the prediction of all-cause death **(a)**, acute coronary syndrome **(b)**, revascularization **(c)** and in the external testing set (n=13,988) for the prediction of all-cause death **(d)**, acute coronary syndrome **(e)** and revascularization **(f)**.

Supplementary Figure 2


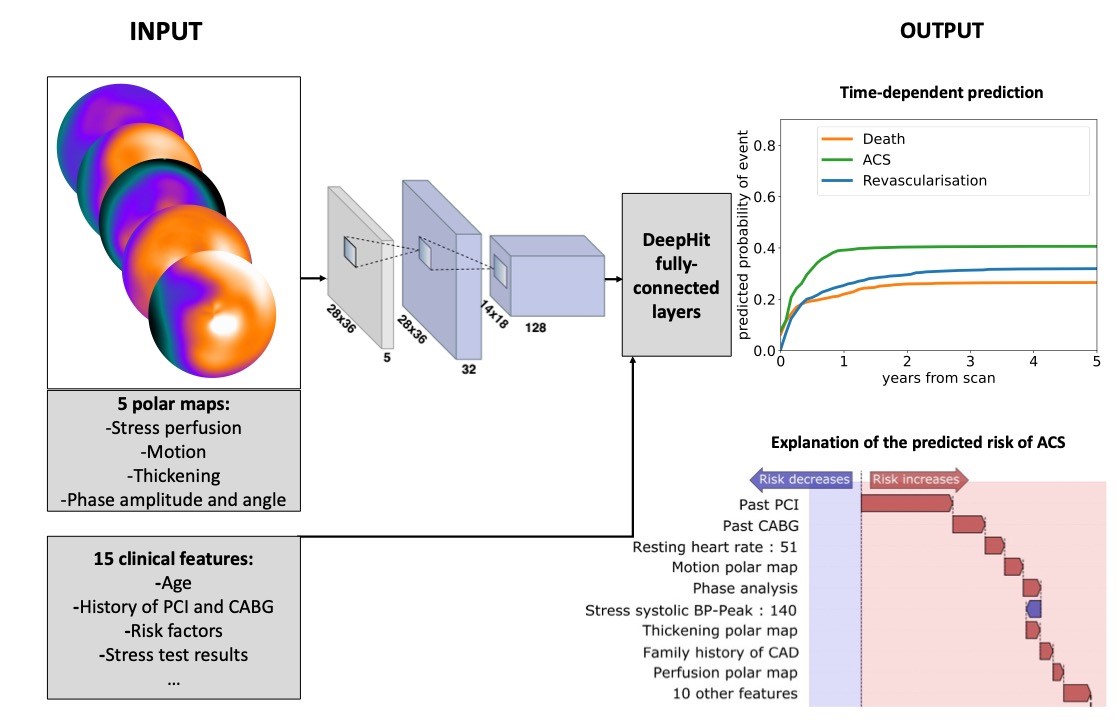


**Model Architecture.** The architecture of proposed model consists of 2 convolution blocks, each with 3x3 convolution kernels, batch normalization, dropout, and Leaky Rectified Linear Unit (ReLU) layers followed by a set of fully connected layers that integrate clinical features into the model and two separate prediction heads (event-specific layers). ACS – acute coronary syndrome, BP – blood pressure, CAD - coronary artery disease, PCI – percutaneous coronary intervention, CABG – coronary artery bypass grafting
