# Supplementary material for: Time and event-specific deep learning for personalized risk assessment after cardiac perfusion imaging
Source: NPJ Digit Med. 2023 May 1;6:78. doi: 10.1038/s41746-023-00806-x (PMC10151323; doi:10.1038/s41746-023-00806-x)
Supplement: Supplementary file 2 — REPORTING SUMMARY [file 41746_2023_806_MOESM2_ESM.pdf]

nature portfolio

Corresponding author(s): Piotr Slomka, PhD, FACC
Director of Innovation in Imaging, Cedars-Sinai
Professor of Medicine and Cardiology
Division of Artificial Intelligence in Medicine, Cedars-Sinai
Professor of Medicine, UCLA School of Medicine
Phone: (310) 423-4348 Fax: (310) 423-0173
Piotr.Slomka@cshs.org

Last updated by author(s): Mar 9, 2023

Reporting Summary

Nature Portfolio wishes to improve the reproducibility of the work that we publish. This form provides structure for consistency and transparency in reporting. For further information on Nature Portfolio policies, see our Editorial Policies and the Editorial Policy Checklist.

Statistics

For all statistical analyses, confirm that the following items are present in the figure legend, table legend, main text, or Methods section.

n/a	Confirmed	
☐	☒	The exact sample size (*n*) for each experimental group/condition, given as a discrete number and unit of measurement
☐	☒	A statement on whether measurements were taken from distinct samples or whether the same sample was measured repeatedly
☐	☒	The statistical test(s) used AND whether they are one- or two-sided<br>*Only common tests should be described solely by name; describe more complex techniques in the Methods section.*
☐	☒	A description of all covariates tested
☐	☒	A description of any assumptions or corrections, such as tests of normality and adjustment for multiple comparisons
☐	☒	A full description of the statistical parameters including central tendency (e.g. means) or other basic estimates (e.g. regression coefficient) AND variation (e.g. standard deviation) or associated estimates of uncertainty (e.g. confidence intervals)
☐	☒	For null hypothesis testing, the test statistic (e.g. *F*, *t*, *r*) with confidence intervals, effect sizes, degrees of freedom and *P* value noted<br>*Give P values as exact values whenever suitable.*
☒	☐	For Bayesian analysis, information on the choice of priors and Markov chain Monte Carlo settings
☒	☐	For hierarchical and complex designs, identification of the appropriate level for tests and full reporting of outcomes
☒	☐	Estimates of effect sizes (e.g. Cohen's *d*, Pearson's *r*), indicating how they were calculated

Our web collection on statistics for biologists contains articles on many of the points above.

Software and code

Policy information about availability of computer code

Data collection	QPS Cardiac Suite (Cedars-Sinai, Los Angeles, CA) was used for imaging data collection and quality control
Data analysis	Python 3.8.12<br>pytorch 1.10.0 - Deep learning framework<br>scikit-learn 0.24.2 - General machine learning framework<br>pycox 0.2.1 - Implementation of DeepHit model loss functions, time-dependent concordance computation<br>pandas 1.2.4 - Data manipulation<br>sklearn-pandas - 2.2.0 Data preprocessing<br>shap 0.40.0 - SHAP values computation and plotting<br>matplotlib - 3.1.1 Plotting<br>scikit-survival - 0.16.0 Cumulative area under receiver operating curve (cAUC) computation<br><br>R 4.1.1<br>dplyr 1.0.7 - Data manipulation

gtsummary 1.4.2 - Table generation, data summary

For manuscripts utilizing custom algorithms or software that are central to the research but not yet described in published literature, software must be made available to editors and reviewers. We strongly encourage code deposition in a community repository (e.g. GitHub). See the Nature Portfolio guidelines for submitting code & software for further information.

Data

Policy information about availability of data

All manuscripts must include a data availability statement. This statement should provide the following information, where applicable:

- Accession codes, unique identifiers, or web links for publicly available datasets
- A description of any restrictions on data availability
- For clinical datasets or third party data, please ensure that the statement adheres to our policy

Provide your data availability statement here.

Human research participants

Policy information about studies involving human research participants and Sex and Gender in Research.

Reporting on sex and gender	Our study considered and reported the sex (biological attribute) only
Population characteristics	The training and internal testing cohort included 20,401 patients undergoing single photon emission computed tomography myocardial perfusion imaging, median age 64 (56, 73), 57% male<br>The external testing set included 13,988 patients from three sites undergoing single photon emission computed tomography myocardial perfusion imaging, median age 67 (59,75), 54% male
Recruitment	All consecutive patients from eight international centers, who underwent single photon emission computed tomography myocardial perfusion imaging between 2009 and 2014
Ethics oversight	This study complies with the Declaration of Helsinki. The institutional review board at Cedars-Sinai as well as at the participating sites approved the collection of data for the registry. Informed consent has been obtained from the subjects (or their legally authorized representative) unless the waiver of consent was granted by the local institutional review board.

Note that full information on the approval of the study protocol must also be provided in the manuscript.

Field-specific reporting

Please select the one below that is the best fit for your research. If you are not sure, read the appropriate sections before making your selection.

☒ Life sciences ☐ Behavioural & social sciences ☐ Ecological, evolutionary & environmental sciences

For a reference copy of the document with all sections, see nature.com/documents/nr-reporting-summary-flat.pdf

Life sciences study design

All studies must disclose on these points even when the disclosure is negative.

Sample size	All consecutive patients from eight international centers, who underwent single photon emission computed tomography myocardial perfusion imaging between 2009 and 2014
Data exclusions	We excluded 17 patients without gated studies, no further exclusions were made
Replication	Model wast trained in a cohort of 20,401 patients (with cross-validation) and then tested in an external cohort of 13,988 patients
Randomization	Data splits for internal cross-validation were random, with stratification for adverse events
Blinding	This is not relevant - the group allocation was only necessary for internal validation of the deep learning model.

Reporting for specific materials, systems and methods

We require information from authors about some types of materials, experimental systems and methods used in many studies. Here, indicate whether each material, system or method listed is relevant to your study. If you are not sure if a list item applies to your research, read the appropriate section before selecting a response.

Materials & experimental systems

n/a	Involved in the study
☒	☐ Antibodies
☒	☐ Eukaryotic cell lines
☒	☐ Palaeontology and archaeology
☒	☐ Animals and other organisms
☐	☒ Clinical data
☒	☐ Dual use research of concern

Methods

n/a	Involved in the study
☒	☐ ChIP-seq
☒	☐ Flow cytometry
☒	☐ MRI-based neuroimaging

Clinical data

Policy information about clinical studies

All manuscripts should comply with the ICMJE guidelines for publication of clinical research and a completed CONSORT checklist must be included with all submissions.

Clinical trial registration	*Provide the trial registration number from ClinicalTrials.gov or an equivalent agency.*
Study protocol	*Note where the full trial protocol can be accessed OR if not available, explain why.*
Data collection	*Describe the settings and locales of data collection, noting the time periods of recruitment and data collection.*
Outcomes	*Describe how you pre-defined primary and secondary outcome measures and how you assessed these measures.*
